# Making the Case for Dental Therapists in the United States

**DOI:** 10.5334/aogh.5050

**Published:** 2026-04-17

**Authors:** Tan Minh Nguyen, Kari Ann Kuntzelman, Eleanor Fleming

**Affiliations:** 1 Deakin Health Economics, Institute for Health Transformation, School of Health and Social Development, Faculty of Health, Deakin University, Melbourne, Australia; 2 Health Economics Group, School of Public Health and Preventive Medicine, Faculty of Medicine, Nursing and Health Sciences, Monash University, Melbourne, Australia; 3 Oral Health Victoria, Melbourne, Australia; 4 American Dental Therapy Association, Executive Director, Wasilla, Alaska, United States; 5 Independent Scholar, Baltimore, United States

## Abstract

Highlighting the historic and contemporary success of dental therapy as an accepted dental provider globally, this manuscript makes the case for the practice in the United States. Co-written with US and Australian practicing dental therapists, this manuscript names a specific challenge from organized dentistry to prevent dental therapists from practicing. Dental therapy is one of the workforce best practices globally and has important implications for the United States. To the degree that best global health practices should be bidirectional, this manuscript aims to make the case for dental therapy in the United States. There is also a call to action for global health advocates to both recognize global oral health challenges and to explore opportunities for collaboration with dental therapists and to elevate best global health workforce practice supporting dental therapy as a certified member of the oral health workforce.

## Introduction

Dental therapy emerged as a new health profession originating from New Zealand, driven by significant community needs from poor oral health among young recruits entering military services during World War I in the early 1920s [1]. Known then as school dental nurses, dental therapists worked under the direct supervision of dentists through school dental services to ensure young recruits had good oral health before enlisting. They provided basic preventive and restorative services within a public health framework [2]. Today, more than 54 countries worldwide have practicing dental therapists [1, 3]. While a dentist’s scope of practice is broadly inclusive of managing health and disease of teeth, gums, and associated structures of the oral cavity, dental therapists have a narrower scope, focused on the prevention and management of dental caries, and managing dental caries with direct restorations among child populations [3, 4]. In some countries like the United Kingdom, regulatory changes have enabled dental therapists’ autonomy, typically referred to as “direct access” [5].

The introduction of dental therapy in New Zealand, Australia, Canada, Hong Kong, Singapore, and Malaysia has demonstrated significant improvements to children’s oral health through increased access and reduced dental caries burden [6]. In 2007, in countries that utilize dental therapists, they make up an estimated 3% of the oral health workforce, compared to 32% being dental hygienists and 65% consisting of dentists [6]. Among the 25 largest countries by population, oral healthcare remains dentist centric, at 2%, 15%, and 83% for dental therapists, dental hygienists, and dentists, respectively [7]. Shifting this oral health workforce skill‑mix is necessary to achieve the World Health Organization’s goal of universal access to essential oral healthcare by 2030 [8]. Given that utilizing dental therapists allows for more cost‑efficient delivery of care, dental therapists are key to realizing optimal oral health for all [9, 10]. While dental therapy has a proven impact globally, the United States has been slow to adopt dental therapy.

The purpose of this manuscript is to explore the historic and contemporary success of dental therapy as an accepted dental provider globally and make the case for the practice in the United States. Because best global health practices should be bidirectional in terms of their impact, this manuscript seeks to apply global health best practices and advocate for dental therapists in the United States.

## The History of Dental Therapy in the United States

In 2004, dental therapy emerged within Alaska Native Communities in the United States as a community‑driven, equity‑centered response to access gaps after witnessing the groundwork set forth in New Zealand to reduce caries rates [11]. The Alaska Dental Therapy Education Program (ADTEP) model reduced barriers to education by removing traditional competitive entry requirements, providing opportunities to a population that has been under‑represented in healthcare [12], and training first‑generation post‑secondary training graduates. Prior to the ADTEP receiving accreditation in 2020 [13] by the Commission on Dental Accreditation (CODA), CODA was not yet accrediting dental therapy programs. However, since its introduction, organized dentistry has consistently opposed this model, invoking concerns about training standards and patient safety. These objections, while often couched in clinical rhetoric, reflect deeper patterns of professional protectionism and systemic racism [14–16]. Dental therapists, in contrast, are designed to serve—not profit from—the community, offering a public health solution grounded in prevention, access, and affordability. As a result of this persistent resistance, according to the American Dental Therapy Association, there are approximately only 210 practicing dental therapists in the United States.

### The oral health problem that requires dental therapy

For over 20 years, the United States has faced oral health disparities with the burden of oral health diseases disproportionately experienced by underserved and vulnerable populations who are historically and contemporarily marginalized and minoritized [17, 18]. As an example, AI/AN children aged three to five years old experience cavities (early childhood caries) at a rate that is five times the prevalence among US children [19, 20]. Across the life span, outcomes such as complete tooth loss continue to demonstrate disparities with minoritized older adults bearing disproportionate impact [21]. The cause of dental diseases is a combination of individual behaviors, such as toothbrushing frequency and diet, and structural and systemic causes of disease that impact the persistence of disease (e.g., community water fluoridation) [22], access to insurance [23], and access to preventative care [24]. However, a primary driver of oral health inequities is the oral health workforce. Regarding access there are two important facts to note: (1) In the United States, 7.5% of the population (24.7 million people) live in dental care shortage areas, which is defined as less than one dentist per 5,000 people in a population; and (2) 1.7 million people in the United States lack access to a dental clinic within a 30‑minute drive [25]. Data from the Health Services Resources Administration Data Warehouse project highlights the persistent gaps in the supply and demand for the oral health workforce [26]. In the United States, the supply of dentists and hygienists fails to meet the demand and population needs, highlighting this country’s oral health workforce shortages. In 2037, the dental hygienist workforce will be 85% adequate and the general dentist workforce 93% adequate for the population demand. In 2025 alone, 10,382 additional oral health providers are needed to address the dental professional shortage areas [27]. To close these gaps, it is worth considering expanding the oral health workforce to include dental therapists.

### The organized dentistry threat

In the United States, the resistance to dental therapy must be viewed through the lens of structural racism and professional exclusivity. Dental therapy is viewed by many as an antiracist solution to oral health inequities [28]. The dental workforce in the United States remains overwhelmingly white and socioeconomically homogenous, while communities with the greatest oral health needs—particularly Black, Indigenous, and Latino populations—are drastically underserved [29–31]. Efforts to expand the dental therapy workforce threaten the economic and control organized dentistry holds over the dental profession [32, 33]. By restricting who can provide care, where care can be delivered, and under what conditions, professional dental organizations maintain control over a lucrative healthcare sector, often at the expense of public need [34–37]. The American Dental Association (ADA) holds the position that despite unequal access to dental care, “a new dental team member is puzzling” [38]. The ADA argues that “dental therapy does not increase access to care in an appropriate, timely and economically feasible way” [39]. In Canada, similar opposition from provincial dental associations led to the closure of publicly funded dental therapy training programs, undermining care access for First Nations populations [40]. By influencing dental boards, legislative bodies, and limiting access to funds, professional dental associations have blocked progress toward integrating dental therapists into the oral health workforce.

Australian dental therapists have traditionally worked in the public sector, delivering oral healthcare solely within the public sector setting of K‑12 schools [41]. The only exception is dental therapists in Western Australia, who also have dental hygiene scope of practice and are not barred from private practice [42]. With changes to the national regulation of the dental profession in 2010 [43], there was harmonization on the core competencies and the scope of practice for all dental practitioner divisions. Due to the increased community need, the dental therapist workforce in Australia is shrinking as dental therapy programs no longer exist in Australia [44]. This is because dental therapy and dental hygiene have similar core competencies, and their combined skillset was rational for a life course approach to managing two most common oral diseases, dental caries and periodontal disease. Hence, oral health therapy in Australia has rapidly expanded, having dual qualifications in dental therapy and dental hygiene. Despite government recommendations made in 2011 [45], dental therapists, dental hygienists, and oral health therapists did not have “direct access” until 2020 [46], meaning that dental therapists could not work autonomously nor independently. However, this change was met with considerable hostility by the Australian Dental Association’s “Hope for Scope” campaign citing “safety” concerns without supporting merit [47]. There is international recognition that a focus on training oral health practitioners, which includes dental therapy, dental hygiene, and oral health therapy, can address global strategies to include oral health into primary care and community settings [48].

Efforts underway in Australia, Canada, and the United States are similar in the ways that organized dentistry has worked against dental therapy. By limiting the scope of practice and naming safety concerns without evidence, organized dentistry has continued to maintain an oral health workforce where power is held in the hands of dentists. To be sure, the position of the ADA, reflective of other dentists organizations, is: “dentists are ultimately responsible, ethically, and legally, for patient care” [49]. Where there are differences in the progress toward advancing dental therapy are due to the number and organization of dental therapists in these countries. Australia has perhaps made the most progress because dental therapy has a longer history. Nevertheless, in the United States, in part because of the decentralized nature of the government, with state laws governing scope of practice, progress is happening with more advocates in states taking steps toward dental therapy [50, 51]. It is important to note that one difference in the United States is the support for dental therapy from dental hygiene professional organizations. The American Dental Hygiene Association supports “oral health care workforce models that cumulate in …. direct access to patient care” [52]. Many of the dental therapy programs in the United States allow the dual licensing in dental hygiene and dental therapy with pathways available for practicing dental hygienists to expand their scope with additional training.

### A call to action for dental therapy

Dental therapy presents an opportunity for an oral health workforce model that functions well in the Global South to expand its reach to the Global North. Despite resistance from professional organizations of some dentists, dental therapy remains a workforce solution to address barriers to dental care. To move forward with dental therapy, policymakers, advocates, professional associations, and communities must support the expansion of dental therapy education and practice in the United States, while also engaging with global oral health efforts to align professional standards, strengthen workforce mobility, and ensure that dental therapists everywhere are empowered to meet community needs and reduce inequities. In addition to the American Dental Therapy Association, in the United States, organizations such as the National Coalition of Dentists for Health Equity, Community Catalyst, a health equity nonprofit organization, and state oral health organizations are vocal proponents for dental therapy. Communities must be allowed to advocate for themselves and for the oral health care professionals that they want.
